# Predicting 30-Day Readmission After Stroke: A Systematic Review and Meta-Analysis to Inform Predictor Selection

**DOI:** 10.3390/diagnostics16111685

**Published:** 2026-05-29

**Authors:** Saurabh Kalra, Farya Fakoori, Mohammad Nafeli Shahrestani, Zhaoqianyu Xiong, Hannah Gardener, WayWay M. Hlaing, Carolina Marinovic Gutierrez, Gillian Gordon Perue, Negar Asdaghi, Jose G. Romano, Tatjana Rundek, Emir Veledar

**Affiliations:** 1 Department of Neurology, University of Miami Miller School of Medicine, Miami, FL 33136, USA; kalra@med.miami.edu (S.K.); fxf288@miami.edu (F.F.); mxn975@med.miami.edu (M.N.S.); zxx374@miami.edu (Z.X.); hgardener@med.miami.edu (H.G.); cgutierrez2@med.miami.edu (C.M.G.); ggordonperue@miami.edu (G.G.P.); nasdaghi@med.miami.edu (N.A.); jromano@med.miami.edu (J.G.R.); trundek@med.miami.edu (T.R.); 2 Department of Public Health Sciences, University of Miami Miller School of Medicine, Miami, FL 33136, USA; whlaing@med.miami.edu

**Keywords:** stroke, 30-day readmission, risk prediction models, prognostic models, machine learning, systematic review, meta-analysis, care transitions, stroke outcomes

## Abstract

**Background:** Thirty-day readmission after stroke remains common, yet contemporary readmission rates, prediction model performance, and predictor domains have not been comprehensively synthesized. **Methods:** Following PRISMA guidelines, we searched PubMed, Embase, Web of Science, Scopus, and Google Scholar for studies published between 1 January 2021 and 9 October 2025. Readmission proportions and model discrimination, measured by area under the receiver operating characteristic curve (AUC), were pooled using random-effects meta-analysis. Heterogeneity was assessed using I^2^. Predictors were summarized across studies by domain. **Results:** Twenty studies met inclusion criteria: 15 studies comprising 358,434 patients contributed quantitative data, and 5 were included in qualitative synthesis only. The pooled proportion was 12.9% (95% CI: 10.1–15.8%), with subgroup estimates of 14.2% (95% CI: 11.9–16.6%) for all-cause and 3.6% (95% CI: 0.5–6.7%) for stroke-specific readmissions. Study-level AUCs ranged from 0.59 to 0.88, with a pooled AUC of 0.69 (95% CI: 0.64–0.75), indicating moderate discrimination. Substantial heterogeneity was observed (I^2^ > 98%, *p* < 0.001), and pooled estimates should be interpreted cautiously. Predictor selection was poorly standardized and largely driven by data availability, with inconsistent inclusion of key clinical and post-discharge domains such as stroke severity, functional status, discharge disposition, post-discharge care, and social determinants of health. **Conclusions:** Thirty-day readmission after stroke remains common, and currently available models demonstrate modest predictive discrimination; no consistently high-performing, broadly generalizable prediction model has yet emerged. Improving prediction will require broader predictors capturing stroke severity, care transitions, follow-up, and patient context, along with external validation and integration into clinical workflows.

## 1. Introduction

Hospital readmission within 30 days after stroke remains a frequent and clinically meaningful outcome, occurring in approximately 10–15% of patients across health systems in the United States and internationally [1,2,3]. Early readmissions are associated with substantial healthcare utilization and cost, fragmented care transitions, and worse patient-centered outcomes, including impaired functional recovery and reduced quality of life [4,5,6]. As a result, 30-day readmission has been widely adopted as a quality metric and target for post-acute care improvement initiatives following discharge from stroke hospitalization [7,8].

Prior observational studies and registry analyses have demonstrated that early readmissions after stroke are often driven by a combination of medical complications, recurrent vascular events, infections, medication-related issues, and challenges in post-discharge care coordination [9,10,11]. Importantly, several studies suggest that a meaningful proportion of readmissions may be potentially preventable, highlighting the need for reliable risk stratification tools to guide transitional care interventions [12,13].

In response, a rapidly expanding body of literature has focused on developing prediction models for 30-day readmission after stroke. These models have leveraged administrative claims, electronic health records, and clinical registry data and have applied both traditional statistical approaches and machine learning techniques [14,15,16,17,18]. However, reported model performance varies widely, and the extent to which these models provide clinically actionable discrimination remains unclear. Many models rely heavily on demographic characteristics and prior healthcare utilization, while clinically important domains such as stroke severity, functional status, social determinants of health, discharge disposition, and post-discharge services are inconsistently incorporated or omitted, potentially limiting model performance and clinical utility [10,15,19].

Prior systematic reviews have examined risk factors for post-stroke readmission (e.g., Deng et al., 2021 [20]) and the performance of prediction models (e.g., Mao et al., 2024 [21]). However, these reviews either focused primarily on individual risk factors without quantitatively evaluating predictive performance or assessed prediction models without jointly synthesizing readmission rates, predictor domains, validation strategies, and contemporary model performance. Although individual cohort studies have explored predictors and causes of post-stroke readmission, no prior systematic review has comprehensively synthesized both readmission burden and pooled discrimination metrics, such as area under the receiver operating characteristic curve (AUC), across contemporary prediction studies. In addition, the extent to which differences in predictor selection contribute to variability in model performance remains incompletely understood [10,11,13,22].

Furthermore, prior work from our group presented at the International Stroke Conference suggested similarly modest performance among earlier readmission prediction studies published before the contemporary electronic health record and machine learning era [23,24,25]. Therefore, the present review focused on studies published from 2021 onward to evaluate whether more recent modeling strategies and data environments have meaningfully improved predictive performance. To address these gaps, we conducted a systematic review and meta-analysis of studies published since 2021 that developed or validated prediction models for 30-day all-cause or stroke-specific readmission after stroke. By pooling readmission proportions and model discrimination metrics and systematically characterizing predictor domains and validation strategies, this study provides a comprehensive assessment of current prediction approaches across three key dimensions: (1) readmission burden, (2) model performance, and (3) predictor selection.

## 2. Methods

### 2.1. Search Strategy and Study Selection

This systematic review and meta-analysis was conducted and reported according to the Preferred Reporting Items for Systematic Reviews and Meta-Analyses (PRISMA) 2020 guidelines [26], and the completed PRISMA checklist is provided in the Supplementary Materials. We systematically searched PubMed, Embase, Web of Science, Scopus, and Google Scholar for studies published between 1 January 2021 and 9 October 2025. Inclusion was restricted to studies published from 2021 onward to provide a focused synthesis of contemporary prediction models developed during the recent expansion of electronic health record integration, machine learning applications, and modern stroke care workflows. The search strategy combined controlled vocabulary and free-text terms related to stroke, 30-day hospital readmission, and prediction or prognostic modeling, including terms for traditional statistical and machine learning approaches (e.g., “stroke,” “hospital readmission,” “30-day,” “predict,” “risk model,” “machine learning,” “logistic regression,” “AUC”). Search syntax was adapted to each database. The complete database-specific search strategies are provided in Table S1. Reference lists of included studies and relevant systematic reviews were manually screened to identify additional eligible records.

All retrieved records were imported into Covidence systematic review software for de-duplication and screening. Two reviewers (SK, FF) independently screened titles and abstracts, followed by full-text reviews of potentially eligible studies. Discrepancies at any stage were resolved through discussion; when consensus could not be reached, a third reviewer adjudicated (MSN). Inclusion criteria were: (1) studies involving adult patients with stroke of any subtype (studies limited to transient ischemic attack (TIA) alone were excluded, though studies including both stroke and TIA were eligible); (2) studies that developed, validated, evaluated, or examined prediction models or predictors for approximately one-month (28–31 days) hospital readmission after discharge; and (3) studies reporting extractable model performance metrics were included for quantitative meta-analysis. Exclusion criteria included gray literature (e.g., dissertations, editorials, commentaries, and preprints without peer review), duplicate publications, non-English language studies, and studies published prior to 1 January 2021. Conference abstracts were included only when sufficient methodological and performance data were available. Outcome definitions varied across studies and included all-cause, unplanned, and stroke-specific readmissions; the handling of planned readmissions, inter-hospital transfers, and 30-day mortality was inconsistently reported.

Exploratory subgroup and univariable meta-regression analyses were additionally conducted to investigate potential sources of heterogeneity, including data source (claims vs. non-claims), inclusion of stroke-severity variables, model type (machine learning vs. traditional statistical approaches), and readmission outcome type. Sensitivity analyses excluding high-performing outlier studies were also performed.

### 2.2. Data Extraction and Quality Assessment

Data extraction was performed independently by four reviewers (SK, FF, MSN, ZX) using a standardized data collection form. Each study was reviewed by at least two extractors, with discrepancies resolved through discussion and consensus among the review team. Extracted variables included first author’s last name, year of publication, country or region, study design and data source, sample size, stroke type, number and proportion of 30-day readmissions, modeling approach (e.g., logistic regression or machine learning), predictor domains, validation strategy, and model performance metrics (area under the receiver operating characteristic curve [AUC] or C-statistic). Predictor variables were grouped into clinically meaningful domains, including demographics, comorbidities, stroke severity, imaging and clinical findings, in-hospital complications, treatments and procedures, functional status, discharge disposition, post-discharge care, prior healthcare utilization before the index stroke hospitalization, and social determinants of health.

When studies reported multiple prediction models, study-level performance estimates were consolidated for quantitative synthesis. Methodological quality and risk of bias were assessed using the Prediction Model Risk of Bias Assessment Tool (PROBAST), which evaluates four domains: participants, predictors, outcome, and analysis [27]. Each domain was rated as having low, high, or unclear risk of bias, and applicability concerns were assessed to evaluate the generalizability of reported model performance. PROBAST ratings are summarized in Table S5 and Figure S2.

In addition to quantitative synthesis, we conducted a structured qualitative synthesis to summarize study-level contributions of included prediction model studies (Table 1) and contextual findings from non-model studies that did not contribute to the meta-analysis (Table S3).

### 2.3. Statistical Analysis

Random-effects meta-analyses were conducted to pool 30-day readmission proportions and model discriminations (AUCs), reflecting substantial between-study heterogeneity. Fixed-effects meta-analyses were performed as sensitivity analyses to assess robustness of pooled estimates. When multiple prediction models were reported within a single study, model-specific AUCs were first combined using inverse-variance weighting to derive a single study-level AUC and standard error, thereby avoiding overweighting non-independent estimates. Between-study heterogeneity was quantified using the I^2^ statistic. Given the expected clinical and methodological heterogeneity across prediction model studies, pooled discrimination estimates were interpreted primarily as descriptive summaries of contemporary model performance rather than precise universal effect estimates. Prespecified subgroup analyses compared model performance for all-cause versus stroke-specific readmissions. Publication bias was assessed through visual inspection of funnel plots and application of the Duval and Tweedie trim-and-fill method [36]. All analyses were performed using Stata version 18.0 (StataCorp LLC, College Station, TX, USA), with inverse-variance methods used to pool proportions and AUC estimates [37]. Outcomes were analyzed separately for all-cause and stroke-specific readmissions where reported. Death within 30 days was not consistently accounted for across studies and was therefore not modeled as a competing risk in pooled analyses. The review protocol was retrospectively registered with the Open Science Framework (OSF) Registries (https://osf.io/qsm3v, accessed on 5 May 2026) to enhance methodological transparency [38].

### 2.4. Ethics Approval

This study was a systematic review and meta-analysis of previously published, de-identified data and did not involve new interactions with human participants or access to identifiable private information. As such, institutional review board approval and informed consent were not required.

## 3. Results

**Figure 1:**
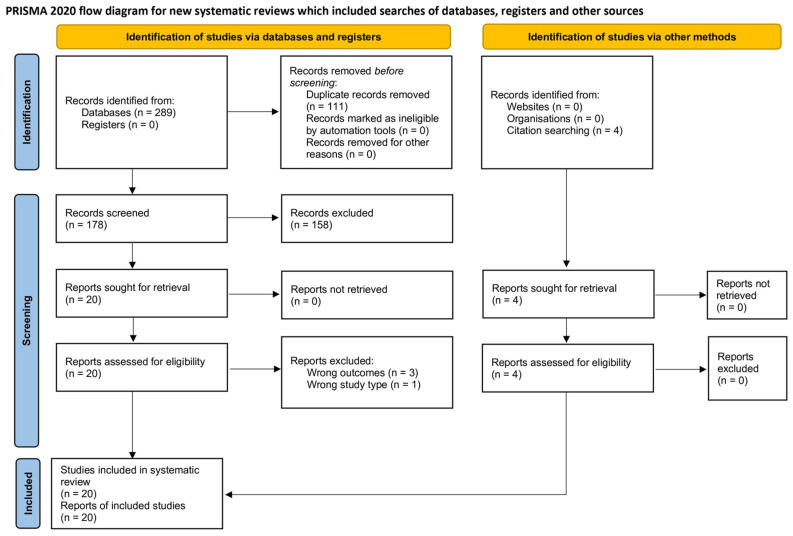
PRISMA 2020 flow diagram of study selection. Of the 20 included studies, 15 contributed quantitative data to the meta-analysis, while 5 were included in qualitative synthesis only. Adapted from Page et al., 2021 [26]. This work is licensed under CC BY 4.0 (https://creativecommons.org/licenses/by/4.0/).

The database search identified 293 records, including 289 records from databases and 4 records from citation searching. After duplicate removal from database records (*n* = 111), 178 records were screened, of which 158 were excluded. Twenty reports from database searches and 4 reports from citation searching were sought for retrieval and assessed for eligibility. Four reports were excluded, leaving 20 studies included in the systematic review. Of these, 15 studies contributed quantitative data to the meta-analysis and 5 were included in the qualitative synthesis only because extractable model performance metrics (e.g., AUC) were unavailable (Figure 1) [10,11,14,15,16,17,18,19,22,28,29,30,31,32,33,34,35,39,40,41]. The included studies were published between 2021 and 2025, and comprised 358,434 patients hospitalized with stroke across diverse geographic regions and data sources. Study characteristics are summarized in Table 1 and Tables S2 and S3.

Reported 30-day readmission proportions differed by outcome definition. Most studies evaluated all-cause readmission (*n* = 13), while only two examined stroke-specific readmissions. Detailed study-level characteristics and model features are presented in Table 1.

At the study level (Table 1), substantial variation in model design, predictors, and performance was observed. A summary of prediction algorithms used across included studies is presented in Table S6. Among higher-performing models, Chen et al. (AUC 0.88) [14] developed an externally validated model using detailed clinical, laboratory, and stroke-severity variables, demonstrating that richer clinical inputs can substantially improve discrimination. Similarly, Hu et al. (AUC 0.84) [28] incorporated functional status and discharge-related variables and achieved relatively strong performance, whereas Ma et al. (AUC 0.82) showed that a well-specified clinical model using routinely available predictors could also achieve strong discrimination, although both relied on internal validation. Lv et al. (AUC 0.80) [17] further supported the contribution of stroke-severity measures such as National Institutes of Health Stroke Scale (NIHSS), whereas Saxena et al. (AUC 0.76) [34] suggested that incorporation of preoperative laboratory variables may improve discrimination, although external validation was lacking.

In contrast, models based primarily on administrative or claims data consistently demonstrated modest performance. Roberts et al. (AUC 0.61) [31] and Kumar et al. (AUC 0.59) [35] showed that even large datasets with extensive comorbidity and utilization variables yielded limited discrimination. Similarly, Nguyen-Huynh et al. (AUC 0.65) found that adding stroke-severity proxies to claims-based models resulted in only modest improvements.

Across multiple studies comparing modeling approaches, machine learning approaches did not consistently outperform traditional regression models. Mercurio et al. (AUC 0.62) [18], Rahmati et al. (AUC 0.60) [22], and Darabi et al. (AUC 0.65) [15] demonstrated minimal differences between logistic regression and advanced machine learning algorithms. High-dimensional approaches incorporating thousands of features, such as Bhaskhar et al. (AUC 0.63) and Lineback et al. (AUC 0.62), similarly did not produce meaningful gains in predictive performance.

Penalized and parsimonious modeling approaches, such as Hailat et al. (AUC 0.68) using LASSO regression, improved model simplicity but did not substantially enhance discrimination. Likewise, simpler models with limited predictors, such as Khan et al. (AUC 0.62), performed comparably to more complex approaches, suggesting that increasing model complexity alone may not meaningfully improve predictive performance.

Across studies, predictor selection varied substantially (Table S4). Most models relied on demographic characteristics, comorbidities, and healthcare utilization prior to the index stroke hospitalization, whereas stroke severity, functional status, discharge disposition, post-discharge care, and social determinants of health variables were inconsistently incorporated. Social determinants of health were infrequently included, limiting assessment of their impact on model performance. External validation was rare (1/15), with most studies relying on internal resampling or split-sample approaches. The lack of external validation further limits confidence in transportability and real-world implementation across healthcare systems.

**Figure 2:**
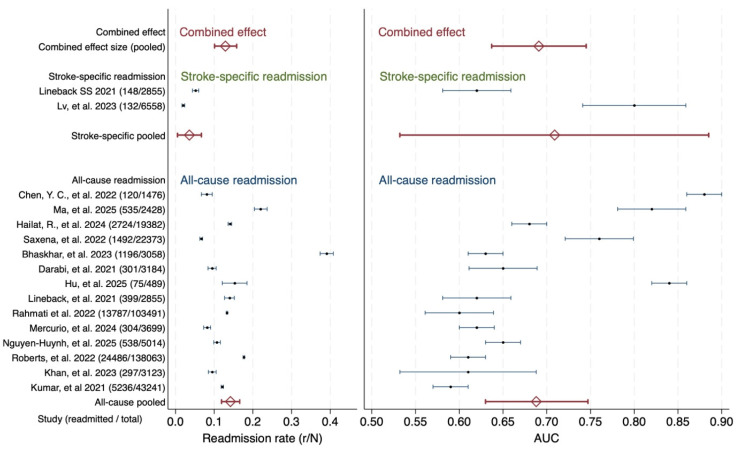
Random-Effects Meta-Analyses for Readmission Proportions and Area Under the Curve (AUC) (*n* = 15) [14,15,16,17,18,19,22,28,29,30,31,32,33,34,35]. Note: Points indicate study-level estimates with 95% confidence intervals. Readmission proportions are shown as readmitted patients/total population (r/N). Diamonds represent random-effects pooled estimates for all-cause, stroke-specific, and combined analyses.

Under random-effects meta-analysis, the overall pooled 30-day readmission proportion was 12.9% (95% CI: 10.1–15.8%), but subgroup estimates were prioritized given differing outcome definitions. Pooled readmission proportions were 14.2% (95% CI: 11.9–16.6%) for all-cause readmissions and 3.6% (95% CI: 0.5–6.7%) for stroke-specific readmissions (Figure 2). Study-level AUCs ranged from 0.59 to 0.88, with a pooled AUC of 0.69 (95% CI: 0.64–0.75); subgroup pooled AUCs were similar for all-cause (0.69; 95% CI: 0.63–0.75) and stroke-specific (0.71; 95% CI: 0.53–0.89) readmission models. Findings should be interpreted cautiously given substantial heterogeneity. Between-study heterogeneity was substantial (I^2^ = 98%, *p* < 0.001). Overall, the pooled AUC of 0.69 indicates that currently available models provide only modest discrimination and that no consistently high-performing, generalizable model for 30-day post-stroke readmission has yet emerged. The wide confidence interval and substantial heterogeneity reflect variability in data sources, predictor domains, patient populations, validation strategies, and outcome definitions.

**Figure 3:**
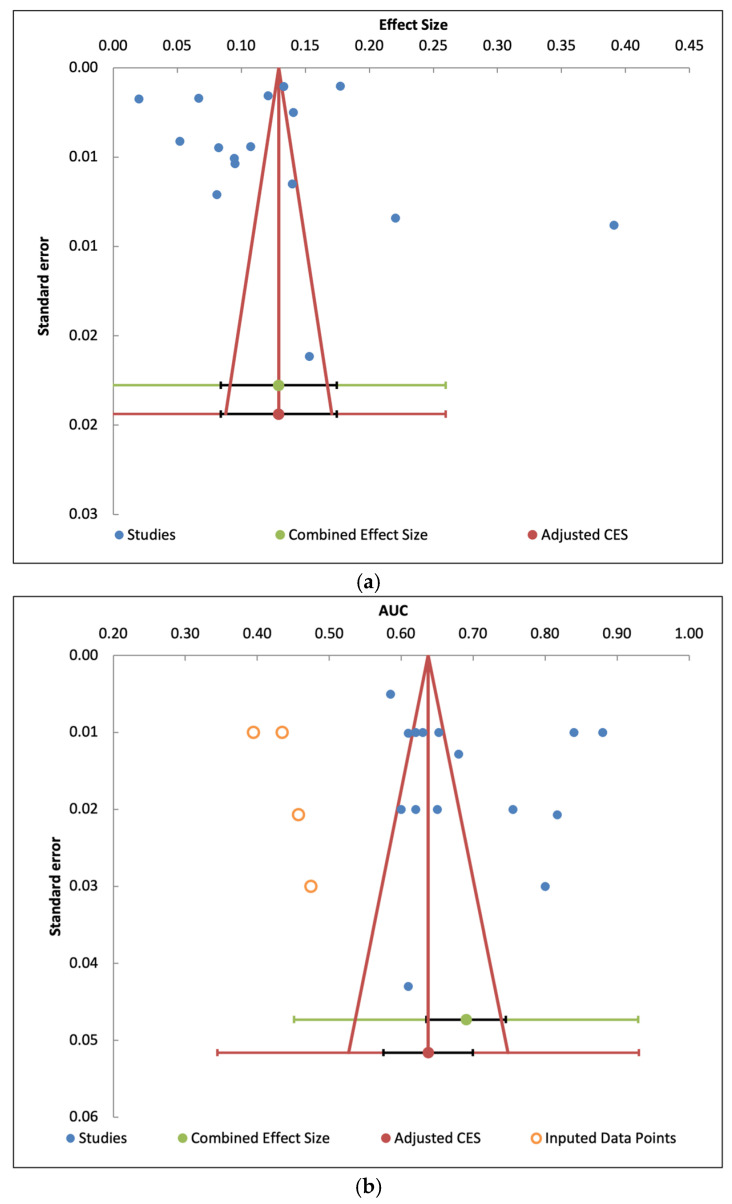
(**a**) Funnel Plot of 30-Day Readmission Proportions. Note: Funnel plot assessing publication bias for study-specific 30-day readmission proportions plotted against standard error. The vertical line represents the pooled readmission proportion under a fixed-effects model. Trim-and-fill-imputed studies and the adjusted pooled estimate are shown. Visual inspection suggests no strong evidence of publication bias. (**b**) Funnel Plot of Model Discrimination (AUC). Note: Funnel plot assessing publication bias for study-level model discrimination measured by area under the receiver operating characteristic curve (AUC). Observed and Trim-and-fill-imputed AUCs are shown along with pooled and adjusted estimates. The largely symmetric distribution suggests limited evidence of publication bias.

Sensitivity analyses using fixed-effects models yielded similar estimates (Figure S1). Most studies were judged to have moderate to high risk of bias, driven primarily by limitations in analytical methods, inadequate handling of missing data, lack of calibration reporting, and reliance on internal validation (Table S5; Figure S2). Funnel plots and trim-and-fill analyses suggested no major publication bias (Figure 3a,b).

Exploratory subgroup and meta-regression analyses were additionally conducted to investigate potential sources of heterogeneity (Table S7). Models incorporating stroke-severity variables demonstrated higher pooled discrimination than models without severity information (AUC 0.738 vs. 0.637). In univariable meta-regression, inclusion of stroke severity was associated with significantly higher model discrimination (β = 0.102, 95% CI: 0.011–0.193; *p* = 0.028), explaining approximately 21.4% of between-study heterogeneity. Claims-based data source, machine learning model type, and readmission outcome type were not statistically significant moderators. Sensitivity analyses excluding high-performing studies (Chen 2022 [14] and Hu 2025 [28]) modestly reduced heterogeneity but did not materially alter pooled estimates, and substantial residual heterogeneity persisted across analyses.

## 4. Discussion

In this systematic review and meta-analysis of contemporary prediction models for 30-day readmission after stroke, we provide a comprehensive assessment across three key dimensions: (1) readmission burden, (2) model performance, and (3) predictor domains. First, we confirm that readmission remains common, affecting approximately one in eight patients. Second, we show that currently available models demonstrate only modest predictive discrimination with substantial heterogeneity. Third, we identify that inconsistent and incomplete representation of clinically meaningful predictor domains is a central limitation underlying current model performance. Importantly, the modest pooled discrimination should be interpreted not only as a limitation of individual algorithms but also as evidence that the field has not yet identified a sufficiently generalizable set of determinants for 30-day post-stroke readmission. In this sense, model performance reflects the current state of knowledge regarding post-stroke rehospitalization risk.

Readmission proportions varied widely across studies, reflecting differences in patient populations (e.g., stroke severity, comorbidity burden, functional status), health-system factors (e.g., discharge disposition, access to post-acute care, care coordination), and outcome definitions [14,31,35]. Given the substantial heterogeneity across studies, pooled estimates should be interpreted as descriptive summaries rather than performance benchmarks. Importantly, 30-day readmission reflects a heterogeneous outcome encompassing both preventable and non-preventable events, including complications, recurrent vascular events, and gaps in care transitions [19,29]. This intrinsic heterogeneity likely constrains achievable discrimination when models rely predominantly on routinely available inpatient data.

Across studies, model discrimination was modest overall, with study-level AUCs ranging from 0.59 to 0.88. This is below the commonly cited threshold of 0.70 for acceptable discrimination [42,43]. Despite the application of diverse machine learning approaches such as random forests, gradient boosting, and neural networks, there was no consistent improvement over traditional regression-based models [15,18,22]. High-dimensional approaches using large electronic health record (EHR) feature sets or natural language processing (NLP)-derived variables also failed to meaningfully improve performance [16,32,42]. These findings suggest that current limitations are driven less by algorithmic sophistication and more by incomplete representation of clinically and socially relevant determinants of post-stroke readmission. Increasing model complexity without improving the relevance of included variables is unlikely to substantially enhance predictive performance. Additionally, machine learning approaches did not consistently outperform traditional logistic regression models across studies. Exploratory subgroup and meta-regression analyses further demonstrated that machine learning model type was not associated with significantly improved discrimination, whereas inclusion of stroke-severity variables was associated with higher model performance and partially explained between-study heterogeneity. This suggests that predictor quality and clinical relevance may be more important determinants of model performance than algorithmic complexity alone.

At the study level, a consistent pattern emerges across included models. Studies incorporating detailed clinical and stroke-severity variables such as Chen et al. (2022) [14], Lv et al. (2023) [17], and Hu et al. (2025) [28] demonstrated relatively higher discrimination, supporting the importance of clinically rich predictors [14,17,28]. In contrast, models based primarily on administrative or claims data (Roberts et al., 2022 [31]; Kumar et al., 2022) [35] consistently showed modest performance despite large sample sizes [31,35]. Models using clinically rich EHR or registry data generally demonstrated better discrimination than those based primarily on claims data. Studies comparing multiple algorithms within the same dataset (Mercurio et al., 2023 [18]; Rahmati et al., 2022 [22]; Darabi et al., 2021) [15] found minimal differences between machine learning and traditional regression approaches [15,18,22]. Importantly, even higher-performing models are not readily generalizable, as they often rely on single-center data, require detailed clinical inputs not consistently available across health systems, and rarely undergo external validation. These limitations restrict their applicability as scalable solutions for routine clinical use.

A central finding of this review is the systematic underrepresentation of key predictor domains. While demographic characteristics, comorbidities, and prior healthcare utilization were nearly universally included across studies [31,35], domains such as stroke severity, functional status, discharge disposition, post-discharge care processes, and social determinants of health were inconsistently incorporated [14,29,33]. This imbalance suggests that predictor selection in currently available models appears driven more by data availability than by clinical or etiologic relevance.

Emerging evidence underscores the importance of these omitted domains. Higher-performing studies tended to incorporate clinically richer variables, including stroke severity, functional status, discharge-related factors, and post-acute care information. This pattern suggests that future improvements are more likely to come from better measurement of post-acute care, social context, and recovery-related factors than from algorithmic complexity alone. Recent work has shown that neighborhood, socioeconomic, and environmental characteristics are independently associated with post-stroke outcomes, highlighting the role of social context [44]. Similarly, disparities in rehabilitation access and functional recovery significantly influence post-stroke outcomes [45]. Post-discharge behaviors and care transitions, including follow-up attendance, medication adherence, and rehabilitation engagement, are also strongly associated with reduced risk of readmission and death [46]. In parallel, explainable machine learning approaches have identified a broad set of relevant predictors, many extending beyond traditional clinical variables [47]. Collectively, these findings suggest that current prediction models are limited by incomplete representation of the multidimensional factors influencing post-stroke recovery and readmission outcomes.

Between-study heterogeneity was substantial and likely reflects real differences in healthcare systems, discharge practices, patient populations, predictor availability, validation strategies, and outcome definitions. Rather than representing only a statistical limitation, this heterogeneity highlights the absence of a broadly transportable prediction framework for 30-day post-stroke readmission. Differences in case mix, health-system structure, predictor definitions, and validation strategies likely contributed to this variability. From an implementation perspective, this heterogeneity constrains adoption of published models without local recalibration or redevelopment incorporating setting-specific predictors, particularly those reflecting transitional care processes and access to post-discharge services [27,48,49]. Variation in outcome definitions may further reduce comparability across studies [43,50].

These observations have important implications. For clinical practice, existing readmission prediction tools, particularly those based on administrative data, are unlikely to provide consistently actionable risk stratification beyond clinical judgment [50]. Efforts to reduce readmissions may therefore benefit more from system-level interventions, including structured transitional care programs, early outpatient follow-up, medical reconciliation, and targeted support for patients with higher social risk [51,52]. For researchers, these findings highlight the need to prioritize data relevance, calibration, transportability, and external validation. Future models should integrate functional status, stroke severity, social risk factors, and post-discharge care processes and should undergo rigorous validation across diverse settings [48,53]. Transparent reporting following PROBAST and TRIPOD guidelines will be essential to improve reproducibility and clinical trust [27,53].

This study has limitations. Substantial heterogeneity reduces precision of pooled estimates, and most included studies were retrospective with moderate to high risk of bias. External validation was uncommon, and calibration was inconsistently reported. Additionally, many studies were conducted in high-income settings, which may limit generalizability. Furthermore, most studies did not capture out-of-hospital deaths within 30 days, which may bias readmission estimates due to competing risks, as death precludes readmission. Many studies also overlapped with the COVID-19 pandemic period, during which changes in healthcare utilization and stroke hospitalization patterns may have influenced readmission estimates.

## 5. Conclusions

Thirty-day readmission after stroke remains common, yet no consistently high-performing and broadly generalizable prediction model currently exists. Across studies, limited discrimination appears to be driven less by modeling approach than by incomplete and inconsistent representation of clinically meaningful predictor domains, including social determinants of health. Most models rely heavily on demographics, comorbidities, and prior healthcare utilization, while key determinants such as stroke severity, functional status, care transitions, post-discharge follow-up, and social context remain underrepresented in the currently available models.

These findings suggest that meaningful improvements in prediction will require broader integration of stroke severity, functional status, post-acute care processes, and social determinants of health rather than increasing algorithmic complexity alone. Until such domains are consistently incorporated and externally validated across settings, currently available models are unlikely to provide reliably actionable risk stratification at the point of discharge.

## Figures and Tables

**Table 1:** Study-Level Contributions of Included Prediction Model Studies (*n* = 15).

Study (Year)	Data Source	Model Type(s)	Key Predictor Domains Included	AUC	Validation Strategy	Main Contribution
Chen (2022) [14]	Hospital EHR (China)	ANN, RF, SVM	Demographics + comorbidities + stroke severity + laboratory + in-hospital clinical variables	0.88	External	One of the few externally validated models with the highest discrimination; highlights the value of detailed clinical and stroke-severity variables
Mercurio (2023) [18]	Hospital EHR (Italy)	LR, RF, XGBoost	Demographics + comorbidities + treatments/procedures + prior utilization	0.62	Internal	ML models do not outperform simpler approaches
Lv (2023) [17]	Registry (China)	XGBoost	Demographics + comorbidities + stroke severity (NIHSS) + in-hospital variables	0.80	Internal	Confirms importance of stroke severity in improving discrimination
Hu (2025) [28]	Hospital EHR (China)	Ensemble ML, RF, ANN	Demographics + comorbidities + stroke severity + functional status + discharge variables + post-discharge care	0.84	Internal	Suggests improved performance when incorporating functional and discharge-related variables
Nguyen-Huynh (2025) [19]	Claims (USA)	LR, RF	Demographics + comorbidities + stroke-severity proxies (NIHSS, mRS) + utilization	0.65	Internal	Severity adds modest improvement in claims-based models
Hailat (2024) [29]	Registry (USA)	LASSO logistic regression	Demographics + comorbidities + stroke severity + in-hospital variables	0.68	Internal	Penalized regression improves parsimony but not discrimination
Khan (2023) [30]	Hospital EHR (USA)	LR	Demographics + comorbidities + prior utilization	0.62	Internal	Simple models perform similarly to complex models
Roberts (2022) [31]	Claims (USA)	LR	Demographics + comorbidities + functional status (FIM) + rehabilitation variables + utilization	0.61	Not reported	Functional status important but overall performance remains modest
Rahmati (2022) [22]	Registry (Iran)	LR, RF, XGBoost	Demographics + comorbidities + behavioral + hospital interventions + utilization + discharge disposition	0.60	Internal	Multiple ML models show minimal performance differences
Bhaskhar (2023) [32]	EHR + audit logs (USA)	ML	High-dimensional EHR features (demographics + comorbidities + utilization + system-level data)	0.63	Internal	Large feature sets do not improve discrimination
Ma (2025) [33]	Hospital EHR (China)	LR	Demographics + comorbidities + stroke severity + in-hospital variables	0.82	Internal	Well-specified clinical model performs comparably to ML
Saxena (2021) [34]	Registry (USA)	RF, NB	Demographics + clinical history + preoperative variables + laboratory values	0.76	Not reported	Incorporation of preoperative laboratory variables was associated with moderate discrimination
Darabi (2021) [15]	Hospital EHR (Iran)	LR, RF, XGBoost	Demographics + comorbidities + stroke severity + clinical variables	0.65	Internal	Confirms similar performance across ML and regression
Kumar (2022) [35]	Claims (USA)	LR	Demographics + comorbidities + claims-based severity proxy (NIHSS) + utilization	0.59	Internal	Claims-based severity insufficient for strong prediction
Lineback (2021) [16]	EHR + NLP (USA)	LR, XGBoost	Demographics + comorbidities + NLP-derived clinical data + utilization	0.62	Internal	NLP increases complexity without improving performance

Abbreviations: LR = logistic regression; ML = machine learning; RF = random forest; XGBoost = extreme gradient boosting; ANN = artificial neural network; NB = naïve Bayes; SVM = support vector machine; LASSO = least absolute shrinkage and selection operator; EHR = electronic health record; NLP = natural language processing; NIHSS = National Institutes of Health Stroke Scale; mRS = modified Rankin Scale; CV = cross-validation; NR = not reported. Note: For studies reporting multiple prediction models, model-specific AUCs were pooled within study using inverse-variance weighting to derive a single study-level estimate used in the meta-analysis.

## Data Availability

No new data were generated for this study. Data used in this systematic review and meta-analysis were extracted from previously published studies cited in the manuscript and Supplementary Materials.

## Supplementary Materials

The following supporting information can be downloaded at: https://www.mdpi.com/article/10.3390/diagnostics16111685/s1, Table S1: Database search strategies; Table S2: Characteristics of Studies Included in the Quantitative Meta-analysis (*n* = 15); Table S3: Characteristics of Studies Included in Qualitative Synthesis Only (*n* = 5); Table S4: Predictor domains reported across included studies (*n* = 20); Table S5: PROBAST risk of bias and applicability assessment (*n* = 15); Table S6: Summary of Prediction Algorithms Across Included Studies; Table S7: Exploratory Subgroup and Meta-Regression Analyses of Model Discrimination (AUC); Figure S1: Fixed-Effect Sensitivity Analysis of Meta-analysis of Readmission Proportions and Area Under the Curve (AUC) (*n* = 15); Figure S2: Risk of Bias Assessment Using PROBAST.

## Abbreviations

The following abbreviations are used in this manuscript:

AUC	Area Under the Curve
CI	Confidence Interval
ED	Emergency Department
HER	Electronic Health Record
I^2^	I-squared (measure of heterogeneity)
LR	Logistic Regression
ML	Machine Learning
NIHSS	National Institutes of Health Stroke Scale
NLP	Natural Language Processing
PRISMA	Preferred Reporting Items for Systematic Reviews and Meta-Analyses
PROBAST	Prediction Model Risk of Bias Assessment Tool
RF	Random Forest
SVM	Support Vector Machine
XGBoost	Extreme Gradient Boosting
ANN	Artificial Neural Network
CV	Cross-Validation
FIM	Functional Independence Measure
mRS	Modified Rankin Scale
NB	Naïve Bayes
NR	Not Reported
TIA	Transient Ischemic Attack
TRIPOD	Transparent Reporting of a Multivariable Prediction Model for Individual Prognosis or Diagnosis
LASSO	Least Absolute Shrinkage and Selection Operator
