# Supplementary material for: Predicting 30-Day Readmission After Stroke: A Systematic Review and Meta-Analysis to Inform Predictor Selection
Source: Diagnostics (Basel). 2026 May 29;16(11):1685. doi: 10.3390/diagnostics16111685 (PMC13256663; doi:10.3390/diagnostics16111685)
Supplement: Supplementary file 1 [file diagnostics-16-01685-s001.zip › diagnostics-4330179-supplementary.pdf]

Supplemental tables/figures

Table S1. Database Search Strategies

Database	Search Strategy
**PubMed**	(“stroke” OR “cerebrovascular accident” OR “CVA”) AND (“hospital readmission” OR “rehospitalization” OR “unplanned readmission”) AND (“30-day” OR “30 day” OR “one month”) AND (“predictive model” OR “prediction model” OR “risk model” OR “risk score” OR “prediction score”) AND (“machine learning” OR “artificial intelligence” OR “deep learning” OR “neural network” OR “random forest” OR “support vector machine” OR “gradient boosting” OR “XGBoost” OR “logistic regression”) AND (“AUC” OR “AUROC” OR “c-statistic”)
**Embase**	(‘stroke’/exp OR ‘cerebrovascular accident’) AND (‘hospital readmission’/exp OR rehospitalization) AND (‘30 day’ OR ‘one month’) AND (‘prediction model’ OR ‘risk model’) AND (‘machine learning’/exp OR ‘artificial intelligence’ OR ‘logistic regression’) AND (‘area under the curve’ OR ‘c statistic’)
**Web of Science**	TS=(stroke OR cerebrovascular accident OR CVA) AND TS=(hospital readmission OR rehospitalization) AND TS=(30-day OR one month) AND TS=(predict* OR risk model OR prediction model) AND TS=(machine learning OR artificial intelligence OR logistic regression OR random forest OR neural network) AND TS=(AUC OR c-statistic)
**Scopus**	TITLE-ABS-KEY(stroke OR cerebrovascular accident OR CVA) AND TITLE-ABS-KEY(hospital readmission OR rehospitalization) AND TITLE-ABS-KEY(30-day OR one month) AND TITLE-ABS-KEY(predict* OR risk model OR prediction model) AND TITLE-ABS-KEY(machine learning OR artificial intelligence OR logistic regression OR random forest OR neural network) AND TITLE-ABS-KEY(AUC OR c-statistic)
**Google Scholar**	“stroke” AND “30-day readmission” AND (“predictive model” OR “risk model” OR “machine learning” OR “logistic regression”) AND (“AUC” OR “c-statistic”)

Note: No date limits were applied in the search strings; publication date restrictions (January 1, 2021 to October 9, 2025) were applied during screening.

Table S2. Characteristics of Studies Included in the Quantitative Meta-analysis (n = 15)

First Author (Year)	Country / Data Source	Sample Size (n)	Readmission Type	No. of Models Evaluated	Primary Model Types	No. of Predictors in Final Model(s)	Validation Strategy	AUC Used for Meta-analysis
Mercurio (2023)	Italy / Hospital EHR	3,699	All-cause	8	LR, DT, RF, XGBoost	12	Internal (5-fold CV)	0.62
Lv (2023)	China / Registry	6,558	Stroke-specific	5	XGBoost	5–20	Internal (5-fold CV)	0.80

First Author (Year)	Country / Data Source	Sample Size (n)	Readmission Type	No. of Models Evaluated	Primary Model Types	No. of Predictors in Final Model(s)	Validation Strategy	AUC Used for Meta-analysis
Hu (2025)	China / Hospital EHR	489	All-cause	11	RF, Stacking, Voting, CatBoost, SVM, GBM, ANN, LR	NR	Internal (bootstrap)	0.84
Chen (2022)	China / Hospital EHR	1,476	All-cause	6	ANN, RF, SVM, KNN, NB, Cox	18	External	0.88
Nguyen-Huynh (2025)	USA / Claims	5,014	All-cause	11	LR, RF	1–6	Internal (5-fold CV)	0.65
Hailat (2024)	USA / Registry	19,382	All-cause	1	LASSO-LR	NR	Internal (split)	0.68
Khan (2023)	USA / Hospital EHR	3,123	All-cause	2	LR	3–8	Internal	0.62
Roberts (2022)	USA / Claims	138,063	All-cause	1	LR	56	None reported	0.61
Rahmati (2022)	Iran / Registry	103,491	All-cause	36	LR, RF, XGB, GBC, NB, AdaBoost	13–31	Internal (10-fold CV)	0.60
Bhaskhar (2023)	USA / EHR + Audit Logs	3,058	All-cause	10	LR, SVM, RF, GBM, ANN	4,812–10,047	Internal (bootstrap)	0.63
Ma (2025)	China / Hospital EHR	2,428	All-cause	1	LR	6	Internal (bootstrap)	0.82
Saxena (2022)	USA / Registry	22,373	All-cause	2	RF, NB	5	NR	0.76
Darabi (2021)	Iran / Hospital EHR	3,184	All-cause	15	LR, RF, XGB, GBM, SVM	14–52	Internal (10-fold CV)	0.65
Kumar (2021)	USA / Claims	43,241	All-cause	4	LR	4–6	Internal	0.59

First Author (Year)	Country / Data Source	Sample Size (n)	Readmission Type	No. of Models Evaluated	Primary Model Types	No. of Predictors in Final Model(s)	Validation Strategy	AUC Used for Meta-analysis
Lineback (2021)	USA / EHR + NLP	2,855	All-cause & Stroke-specific	16	LR, XGB, Ensemble	NR	Internal (CV/Bootstrap)	0.62

Abbreviations: LR = logistic regression; DT = decision tree; RF = random forest; XGB = XGBoost; GBM = gradient boosting machine; ANN = artificial neural network; NB = naïve Bayes; SVM = support vector machine; KNN = k-nearest neighbors; LASSO = least absolute shrinkage and selection operator; GBC = gradient boosting classifier; Cox = Cox proportional hazards model; AdaBoost = adaptive boosting; CatBoost = categorical boosting; CV = cross-validation; NR = not reported.

Note: For studies reporting multiple prediction models, model-specific AUCs were pooled within study using inverse-variance weighting to derive a single study-level estimate used in the meta-analysis.

Table S3. Characteristics of Studies Included in Qualitative Synthesis Only (n = 5)

First Author (Year)	Country	Study Design	Sample Size (n)	Stroke Type	30-Day Readmission Rate	Primary Focus	Key Findings
Bondi (2021)	USA	Retrospective cohort	NR	Stroke (all types)	NR	Predictors	Identified comorbidity burden and discharge disposition as major drivers of early readmission
Gardener (2023)	USA	Prospective cohort	NR	Ischemic stroke	NR	Predictors	Identified clinical, functional, insurance, stroke-care, and discharge factors associated with 30-day readmission
Jun-O'Connell (2022)	USA	Retrospective cohort	NR	Stroke (all types)	NR	Risk score evaluation	LACE+ score was associated with unplanned 30-day readmission after stroke
Loebel (2022)	USA	Retrospective cohort	NR	Stroke (all types)	NR	Predictors	Identified NIHSS ≥5, longer length of stay, CAD, and prior stroke/TIA as

First Author (Year)	Country	Study Design	Sample Size (n)	Stroke Type	30-Day Readmission Rate	Primary Focus	Key Findings
							predictors of 30-day readmission
Liu (2022)	China	Retrospective cohort	NR	Acute ischemic stroke	NR	Predictors & Costs	Identified demographic, insurance, hospital-level, length of stay, and comorbidity factors associated with 30-day readmission and cost

Note: These studies did not report extractable discrimination metrics suitable for quantitative synthesis; therefore, they were excluded from meta-analysis but included to contextualize findings. Abbreviations: AUC = area under the curve; CAD = coronary artery disease; NIHSS = National Institutes of Health Stroke Scale; NR = not reported; TIA = transient ischemic attack; LACE+ = length of stay, acuity of admission, comorbidities, emergency department use, and additional patient/hospital factors.

Table S4. Predictor Domains Reported Across Included Studies (n = 20)

Study (First Author, Year)	Demographics	Comorbidities	Stroke Severity	Imaging / Clinical Findings	In-hospital Complications	Procedures / Treatments	Functional Status	Discharge Disposition	Post-discharge Care	Prior Utilization	Social Determinants of Health
**Quantitative synthesis (n = 15)**											
**Mercurio 2023**	✓	✓	–	–	✓	✓	–	✓	–	✓	–
**Lv 2023**	✓	✓	✓ (NIHSS)	–	✓	✓	–	✓	–	✓	–
**Hu 2025**	✓	✓	✓	–	✓	✓	✓	✓	✓	✓	–
**Chen 2022**	✓	✓	✓	✓	✓	✓	–	✓	–	✓	–
**Nguyen-Huynh 2025**	✓	✓	✓ (NIHSS, mRS)	–	–	✓	✓	✓	–	✓	–
**Hailat 2024**	✓	✓	✓	–	✓	✓	–	✓	–	✓	–

Study (First Author, Year)	Demographics	Comorbidities	Stroke Severity	Imaging / Clinical Findings	In-hospital Complications	Procedures / Treatments	Functional Status	Discharge Disposition	Post-discharge Care	Prior Utilization	Social Determinants of Health
**Khan 2023**	✓	✓	–	–	–	–	–	–	–	✓	–
**Roberts 2022**	✓	✓	–	–	–	✓	✓	✓	–	✓	–
**Rahmati 2022**	✓	✓	✓	–	✓	✓	–	✓	–	✓	–
**Bhaskhar 2023**	✓	✓	–	–	✓	✓	–	✓	–	✓	–
**Ma 2025**	✓	✓	✓	–	✓	✓	–	✓	–	✓	–
**Saxena 2022**	✓	✓	–	–	–	✓	–	✓	–	✓	–
**Darabi 2021**	✓	✓	✓	✓	✓	✓	–	✓	–	✓	–
**Kumar 2021**	✓	✓	✓ (claims-based NIHSS)	–	–	–	–	–	–	✓	–
**Lineback 2021**	✓	✓	–	✓ (NLP)	✓	✓	–	✓	–	✓	–
**Qualitative synthesis only (n = 5)**											
**Bondi 2021**	✓	✓	–	–	✓	✓	–	✓	–	✓	–
**Loebel 2022**	✓	✓	✓	–	–	–	–	–	–	✓	–
**Jun-O'Connell 2022**	✓	✓	–	–	–	–	–	–	–	✓	–
**Gardener 2023**	✓	✓	✓	–	✓	✓	–	✓	–	✓	–

Study (First Author, Year)	Demographics	Comorbidities	Stroke Severity	Imaging / Clinical Findings	In-hospital Complications	Procedures / Treatments	Functional Status	Discharge Disposition	Post-discharge Care	Prior Utilization	Social Determinants of Health
**Liu 2022**	✓	✓	–	–	–	–	–	–	–	✓	–

Note: Variables represent major predictor domains reported at the study level; individual models within studies may include additional predictors. A dash indicates that the domain was not reported or not clearly included as a predictor. No included studies explicitly incorporated social determinants of health as structured predictors. Abbreviations: NIHSS = National Institutes of Health Stroke Scale; mRS = modified Rankin Scale; NLP = natural language processing.

Table S5. PROBAST risk of bias and applicability assessment (n = 15)

Study (First Author, Year)	Participants	Predictors	Outcome	Analysis	Overall Risk of Bias
Chen 2022	Low	Low	Low	**Low**	**Low**
Mercurio 2023	Low	Low	Low	**High**	**High**
Lv 2023	Low	Low	Low	**High**	**High**
Hu 2025	Low	Low	Low	**High**	**High**
Nguyen-Huynh 2025	Low	Low	Low	**High**	**High**
Hailat 2024	Low	Low	Low	**High**	**High**
Khan 2023	Low	Low	Low	**High**	**High**
Roberts 2022	Low	Low	Low	**High**	**High**
Rahmati 2022	Low	Low	Low	**High**	**High**
Bhaskhar 2023	Low	**Unclear**	Low	**High**	**High**
Ma 2025	Low	Low	Low	**High**	**High**
Saxena 2022	Low	Low	Low	**High**	**High**
Darabi 2021	Low	Low	Low	**High**	**High**
Kumar 2021	Low	Low	Low	**High**	**High**
Lineback 2021	Low	**Unclear**	Low	**High**	**High**

Note: Overall risk of bias was rated as high if any PROBAST domain was judged high risk. High risk in the analysis domain predominantly reflects incomplete reporting or suboptimal methods for overfitting control, missing data handling, calibration, and reliance on internal validation without independent external validation.

Table S6. Summary of Prediction Algorithms Across Included Studies

Algorithm Type	Number of Studies	General Observation
Logistic regression	9	Most commonly used traditional modeling approach
Random forest	7	Frequently used machine learning method
XGBoost/boosting methods	6	Commonly applied in higher-dimensional datasets
ANN/neural networks	3	Limited external validation
NLP/high-dimensional ML	2	Minimal improvement in discrimination

Table S7. Exploratory Subgroup and Meta-Regression Analyses of Model Discrimination (AUC)

**Analysis**	**No. Studies**	**Pooled AUC (95% CI)**	**I^2 (%)**	**Meta-regression p-value**
Overall	15	0.691 (0.640–0.742)	98.86	—
**Data source**				
Claims-based studies	3	0.616 (0.581–0.650)	93.89	0.126
Non-claims studies	12	0.710 (0.651–0.769)	97.91	
**Stroke severity variables**				
Stroke severity included	8	0.738 (0.663–0.814)	99.13	0.028
No stroke severity variables	7	0.637 (0.595–0.678)	93.19	
**Modeling approach**				
Machine learning models	10	0.705 (0.640–0.770)	98.38	0.447
Traditional/statistical models	5	0.662 (0.579–0.746)	98.54	
**Readmission outcome type**				
All-cause readmission	13	0.688 (0.633–0.744)	99.01	0.864
Stroke-specific readmission	2	0.710 (0.530–0.890)	95.42	
**Sensitivity analysis**				
Excluding Chen 2022 and Hu 2025	13	0.664 (0.622–0.705)	97.78	—

Abbreviations: AUC = area under the receiver operating characteristic curve; CI = confidence interval; ML = machine learning.

Note: Exploratory subgroup and univariable random-effects meta-regression analyses were conducted to investigate potential sources of heterogeneity. Stroke severity variables included NIHSS, mRS, or other severity-related clinical indicators. Meta-regression p-values correspond to between-group comparisons.

Figure S1. Fixed-Effect Sensitivity Analysis of Meta-analysis of Readmission Proportions and Area Under the Curve (AUC) (n = 15).

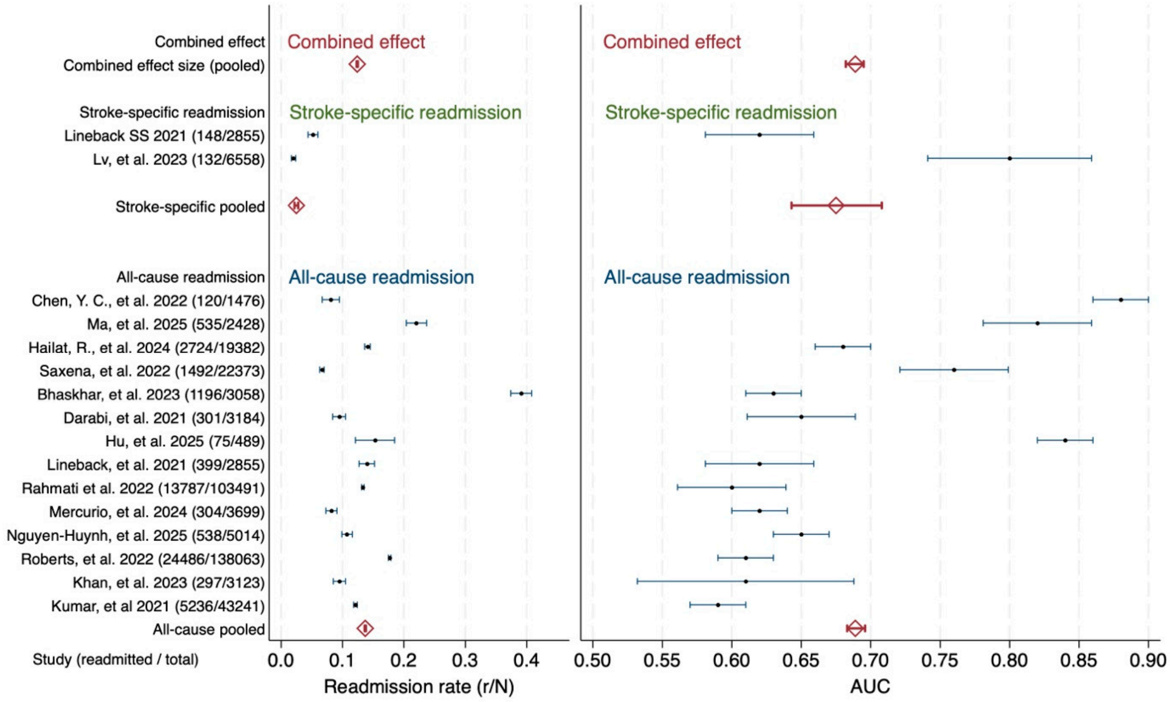

Note: Sensitivity analysis using fixed-effects meta-analysis. Points represent study-level estimates with 95% confidence intervals. Diamonds represent random-effects pooled estimates for all-cause, stroke-specific, and combined analyses. Results were compared with the primary random-effects models to assess robustness of pooled estimates to model specification.

Figure S2. Risk of Bias Assessment Using PROBAST.

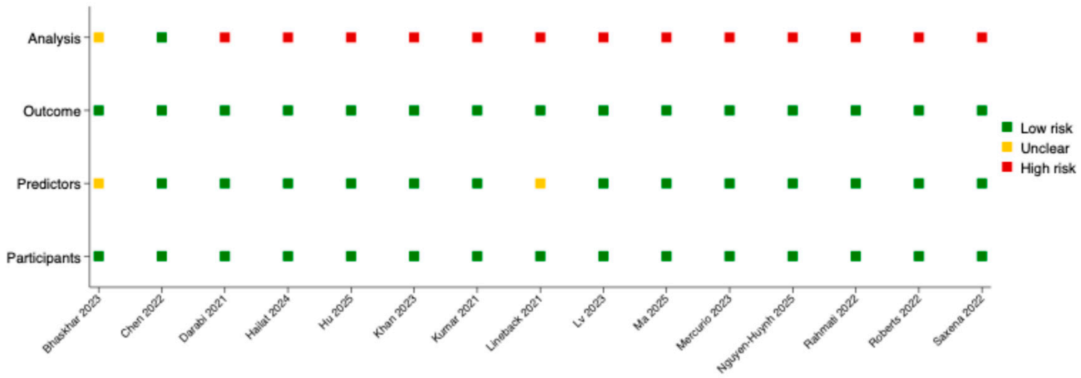

Traffic-light plot summarizing risk of bias across PROBAST domains. Green indicates low risk, yellow unclear risk, and red high risk. High risk in the analysis domain predominantly reflects reliance on internal validation without independent external validation.
